# Prevalence of signs of lower urinary tract disease and positive urine culture in dogs with diabetes mellitus: A retrospective study

**DOI:** 10.1111/jvim.16634

**Published:** 2023-01-28

**Authors:** Valerie Nelson, Amy Downey, Stacie Summers, Sarah Shropshire

**Affiliations:** ^1^ Colorado State University Veterinary Teaching Hospital Fort Collins Colorado USA; ^2^ Davis Veterinary Medical Teaching Hospital University of California Davis California USA; ^3^ Oregon State University Veterinary Teaching Hospital Corvallis Oregon USA

**Keywords:** antibiotics, bacteriuria, canine, urinalysis, urinary tract infection

## Abstract

**Background:**

No recent studies have evaluated the association between clinical signs of lower urinary tract disease (LUTD) and positive urine culture in dogs with diabetes mellitus.

**Objective:**

Determine the prevalence of subclinical bacteriuria (ie, positive urine culture without signs of LUTD) in dogs with diabetes mellitus.

**Animals:**

One hundred seven dogs with diabetes mellitus were evaluated at a university veterinary hospital.

**Methods:**

Retrospective study evaluating diabetic dogs with a single sample paired urinalysis and urine culture. Relationship between the presence of signs of LUTD, pyuria, and bacteriuria and urine culture results were compared using Fisher exact testing.

**Results:**

Fifteen dogs (14%) had a positive urine culture via cystocentesis or free catch, of which 8 (53%) had pyuria, and 4 (27%) had signs of LUTD. Of the 88 dogs (82%) without signs of LUTD, 11 (13%) had a positive culture. A significant association was found between a positive urine culture and pyuria (OR infinity; 95% CI 20.34‐infinity, *P* < .00001) and bacteriuria (OR infinity; 95% CI 164.4‐infinity, *P* < .00001). No association was found between urine culture results and signs of LUTD (OR 1.87; 95% CI 0.59‐6.85, *P* = .46).

**Conclusion and Clinical Importance:**

Subclinical bacteriuria occurred in this cohort of dogs, and our findings reinforce the recommendation that urine cultures should not be routinely performed in diabetic dogs particularly if pyuria and bacteriuria are absent.

**Abbreviations**
AB: asymptomatic bacteriuria
FOV: field of view
hpf: high‐powered field
IVDD: intervertebral disc disease
lpf: low‐powered field
LUTD: lower urinary tract disease
PD: polydipsia
PU: polyuria
RBC: red blood cell
SB: subclinical bacteriuria
SSA: sulfosalicylic acid
USG: urine specific gravity
USMI: urethral sphincter mechanism incompetence
UTI: urinary tract infection
WBC: white blood cell

## 1 INTRODUCTION

Recommendations for when to submit urine cultures in diabetic dogs have dramatically changed over the last 25 years. Previously, routine submission of urine cultures was recommended and commonly performed in diabetic dogs regardless of clinical signs or urinalysis results.^1^, ^2^ Reasons for this recommendation included concerns for infection promoting insulin resistance and diabetic ketoacidosis, increased risk for urinary tract infections because of altered immune function of immune cells such as granulocytes, and these potential lower urinary tract infections serving as a source of septicemia if left untreated.^1^, ^2^, ^3^ In human medicine, a urinary tract infection (UTI) in a diabetic patient was categorized as a complicated UTI and this verbiage was initially adopted in veterinary medicine.^4^ Studies in dogs show that a positive urine culture can occur without signs of lower urinary tract disease (LUTD) or expected urine sediment changes such as pyuria or cytologic presence of bacteria.^1^, ^2^, ^5^ Therefore, screening urinalyses and urine cultures in diabetic dogs were commonly performed in general and specialty practice.

Over time these recommendations have changed to mirror current human guidelines and to support better antimicrobial stewardship in veterinary medicine. Consensus guidelines for antimicrobial use in animals discouraged the submission of urine cultures in dogs that are not showing signs of lower urinary tract disease.^6^ Expected signs of LUTD associated with a UTI could include stranguria, pollakiuria, dysuria, malodorous urine, hematuria, inappropriate urination, or a combination of these signs.^7^, ^8^ The consensus guidelines also recommended to not treat subclinical bacteriuria (SB). Subclinical bacteriuria is defined as a positive bacterial urine culture in an animal that is not showing any clinical signs of urinary tract disease.^8^ In humans, asymptomatic bacteriuria (AB) is a common finding, particularly in healthy women or individuals with concurrent urologic abnormalities.^9^ Screening for AB is only recommended in certain clinical scenarios such as in pregnant women or in patients undergoing invasive endourologic procedures. Screening or treating for AB is not recommended in people with diabetes mellitus.^9^

Subclinical bacteriuria under the current definition also occurs in healthy dogs^5^, ^10^, ^11^ and is documented in diabetic dogs.^1^, ^2^ Despite the aforementioned guidelines, in diabetic dogs lacking clinical evidence of active lower urinary disease, the finding of inflammatory changes or cytologic evidence of bacteria in the urine, a concurrent positive bacterial culture, or a combination of these findings can make veterinarians uneasy if left untreated. However, treating dogs that have subclinical bacteriuria can contribute to antimicrobial resistance, increase cost to the owner, affect the human and animal bond during medication administration, and potentially result in adverse effects because of the prescribed antimicrobial.

With these newer changes in guidelines, there have been no recent studies in diabetic dogs evaluating the presence of signs of LUTD and subclinical bacteriuria under the current definitions. Therefore, the purpose of this study was to determine the prevalence of subclinical bacteriuria in diabetic dogs. Our hypothesis was that subclinical bacteriuria is common in diabetic dogs reinforcing the current guidelines that urine cultures should only be submitted when clinically indicated.

## 2 MATERIALS AND METHODS

### 2.1 Medical record review

This was a retrospective study that evaluated medical records of dogs with diabetes mellitus that were presented to Colorado State University Veterinary Teaching Hospital between January 1, 2008, and January 1, 2019. All owners signed the standard intake form upon admission which approves the use of data reporting for research purposes. Dogs were diagnosed with diabetes mellitus based on consistent clinical signs (ie, polyuria (PU), polydipsia (PD), ± weight loss with polyphagia), and hyperglycemia (>300 mg/dL) with glucosuria. Signs of LUTD were considered present if stranguria, pollakiuria, dysuria, hematuria, licking at the vulva in the absence of discharge or urinary incontinence, or inappropriate urination were reported by the owners.

Initial screening criteria of medical records included an initial paired urinalysis and urine culture in a dog with diabetes mellitus. Urine cultures that were submitted greater than 3 days after the urinalysis submission were excluded. Exclusion criteria at initial evaluation included suspected or confirmed hyperadrenocorticism, hypothyroidism, azotemic chronic kidney disease, systemic hypertension, diabetic ketoacidosis, urolithiasis, urinary polyps, lower urinary system neoplasia, intervertebral disc disease (IVDD), a previous diagnosis of or receiving medications for urethral sphincter mechanism incompetence (USMI), abnormal vulvar conformation, vaginal discharge, diagnosis of vaginitis, ectopic ureters, nonambulatory state, concurrent daily steroid administration at any dose or route, and immunosuppressive treatment. Dogs were also excluded if they had been treated with an antimicrobial within 60 days of the urine collection. Antimicrobials to treat diarrhea (ie, tylosin, metronidazole) within 60 days of urine collection were not considered a reason for exclusion.

Data gathered from the medical record included age, breed, sex, body weight, presence of signs of LUTD, urinalysis, urine culture, current, and recent medications. Information specific to the urinalysis included the date of submission, route of collection, urine specific gravity, pH, protein, and glucose on urine dipstick, cytology findings (white blood cells [WBCs], red blood cells [RBCs], bacteria, casts). Sulfosalicylic acid test (SSA) results were recorded, if performed. Classification and quantification were recorded for applicable parameters (crystals, casts, WBCs, RBCs, etc.). Pyuria was defined as >6 WBC/high powered field (hpf) in a non‐hematuric (defined as <250 RBC/hpf) urine sample. Similarly, urine culture results recorded were the date submitted, aerobic culture results, anaerobic culture results, colony forming units (cfu) per mL of bacteria present, and if and which antimicrobial was prescribed. Additionally, any available follow‐up information after the initial visit was recorded. If the route of urine collection was not listed within the medical record and could not be determined, the sample was assigned to the voided group. The definitions used to describe WBC and RBC numbers are the following: rare and trace were considered equivalent terminology, rare was defined as 1 to 10 cells on the entire slide for both WBCs and RBCs, occasional was defined as 0 to 5 cells per hpf for RBCs and 0 to 3 cells per hpf for WBCs, and packed field was defined as many cells were present where the determination of an actual number was not possible. During the study timeframe, the laboratory changed how casts were reported. For the purposes of the study, both classifications (descriptive and numerical) were recorded. For the descriptive classifications, the terms were defined as the following: few casts were defined as 0% to 10% per hpf, difficult to find on the slide, and up to one or more in almost every field; moderate casts were defined as 10% to 50% per hpf, easy to find, and the number present in the field of view (FOV) varies; and many casts were defined as >50% per hpf with a large number present on all FOVs. For the numerical classifications, 0 to 1, 1 to 3, 3 to 6, 6 to 10, and >10 casts present per low power field (lpf) were recorded.

Routine urinalysis with sediment examination was performed by the clinical pathology service with at least 5 mL of freshly submitted refrigerated urine within a few hours as described.^12^ At least a 1 mL aliquot of urine was submitted for urine culture to the Colorado State University Veterinary Diagnostic Laboratory.

### 2.2 Statistical analysis

Descriptive statistics were reported for population data and various urinalysis and urine culture data. A Fisher exact test was performed to evaluate if there was a difference between proportions of dogs based on urine culture results (positive or negative) and the presence or absence of signs of lower urinary tract disease. The same analysis was performed to evaluate if there was a difference between proportions of dogs based on urine culture results (positive or negative) and whether there was cytologic presence of bacteria or pyuria detected on urine sediment microscopy. Statistical analysis was performed using GraphPad Prism version 9 with a Fisher's exact test and was considered significant if *P* < 0.05.

## 3 RESULTS

### 3.1 Animals

This study initially contained 657 potential paired urinalysis and culture samples for evaluation but after screening for exclusion criteria, 107 dogs were included for evaluation. Median age was 9 years old (range 14 weeks‐15 years). Breeds with multiple dogs represented included mixed breed (30/107, 28%), Labrador Retriever (12/107, 11.2%), Miniature Schnauzer (6/107, 5.6%), Bichon Frise (4/107, 3.7%), West Highland White Terrier (4/107, 3.7%), Rottweiler (4/107, 3.7%), Cairn Terrier (3/107, 2.8%), Dachshund (3/107, 2.8%), and Miniature Poodle (3/107, 2.8%). Reproductive status was 47.7% male castrated (51/107), 44.9% female spayed (48/107), 4.7% female intact (5/107), and 2.8% male intact (3/107). Weight was assigned to one of 3 categories 0 to 10 kg (44/107, 41.1%), 10.1 to 40 kg (52/107, 48.6%), or >40.1 kg (11/107, 10.3%).

### 3.2 Urinalysis

The route of urine collection was predominantly via cystocentesis (96/107, 89.7%) and the remaining samples were collected from voided urine. All samples were refrigerated and processed by the diagnostic laboratory within a few hours, and all were cultured on‐site as per our standard protocol. Urine specific gravity (USG) ranged from 1.006 to 1.073, with a median value of 1.033. The urine pH minimum was 5.0 and the maximum was 9.0, with a median value 6.4.

Crystalluria was rare and included amorphous 5.6% (6/107), calcium oxalate dihydrate 4.7% (5/107), and struvite 1.9% (2/107) crystals. In the 2 dogs with struvite crystalluria, neither dog demonstrated signs of LUTD and the urine cultures were both negative. Granular (11.2%, 12/107) casts were found in a subset of the study population (rare, 41.6%, 5/12; occasional, 58.3%, 7/12).

Proteinuria was present in 57.9% of samples (62/107) as defined by dipstick analysis. Samples were considered proteinuric if urine dipstick reading was >1+. Most samples with proteinuria had trace protein (26.2%, 28/107). Samples with proteinuria of 1+ was 15.9% (17/107), of 2+ was 10.3% (11/107), and of 3+ was 5.6% (6/107). SSA was performed in most of the samples (81.3%, 87/107). Of samples that had SSA performed, 44% (38/87) of the time protein was not documented. SSA detected protein in trace quantities 17% (15/87), 1+ 22% (19/87), 2+ 10% (9/87), 3+ 6% (5/87), and 4+ 1% of the time (1/87).

Glucosuria was present in 71.0% (76/107) of samples. The prevalence of trace glucosuria was 4.7% (5/107), 1.9% had 1+ glucosuria (2/107), 2.8% had 2+ glucosuria (3/107), 8.4% had 3+ glucosuria (9/107), and 53.3% had 4+ glucosuria (57/107).

Cytologic presence of bacteria was noted in 15% of samples (16/107). When the number of bacteria was quantified into groups, 2 samples were recorded as “few,” 6 samples were recorded as “moderate,” and 7 were recorded as “many.” In one sample, the number of bacteria was not quantified. Most samples showed bacteria with rod morphology (15/16) and one sample showed cocci on cytology (1/16).

Pyuria was present in 7.5% (8/107) of the urine samples. Two samples (1.9%, 2/107) were considered hematuric (>250 RBC/hpf).

### 3.3 Urine culture

Most urine samples (94.4%) were submitted for aerobic culture (101/107) with the remaining 5.6% submitted for aerobic and anaerobic culture (6/107). Urine cultures were submitted on the same day as the urinalysis for 62.6% (67/107) of the dogs with the remaining samples being submitted 1 day (28%, 30/107), 2 days (6.5%, 7/107), or 3 days later (2.8%, 3/107).

Urine cultures were positive in 15 dogs (14%, 15/107). The organisms that were isolated included *Escherichia coli* (80%, 12/15), *Enterobacter aerogenes* (7%, 1/15), *Enterococcus* spp., (7%, 1/15), and *Pseudomonas aeruginosa* (7%, 1/15). One culture grew *Aspergillus* spp., which was considered contaminated by the clinical pathology lab and not a true positive. As such, this urine sample was reported in the “No Growth” category. Of the samples that cultured *E*. *coli*, 5 samples isolated *E*. *coli* hemolytic and one isolated an equal mixture of both colonies (ie, *E*. *coli* and *E*. *coli* hemolytic). In the samples that cultured bacteria, the majority had >100 000 cfu/mL (46.7%, 7/15). Of those 7 urine cultures, 6 grew a single isolate at 100000 cfu/mL. The remaining samples grew 2 isolates with each measuring 100 000 cfu/mL. The remaining bacteria counts were between 10 000 to 100 000 cfu/mL in 33.3% (5/15) and less than or equal to 5000 cfu/mL in 20% (3/15) of the samples.

Of the 15 samples which yielded a positive urine culture, 15 (100%) displayed the cytologic presence of bacteria. Only one sample which yielded a negative urine culture (1%), displayed the cytologic presence of bacteria. Note: this sample was obtained via cystocentesis and was submitted for culture within 24 hours from collection. There was a significant association found between urine culture and bacteriuria (OR infinity; 95% CI 164.4‐infinity, *P* < .00001).

Of the 15 samples with a positive urine culture, 8 (53%) were found to be pyuric. No samples yielding a negative urine culture were found to be pyuric. There was a significant association found between urine culture and pyuria (OR infinity; 95% CI 20.34‐infinity, *P* < .00001).

Of the 107 samples, 96 (90%) were obtained via cystocentesis, and 11 (10%) were collected via voided sample. Of those collected via cystocentesis, 11 (11%) yielded a positive urine culture. Of those obtained via voided sample, 4 (36%) yielded a positive urine culture (these 4 samples were submitted for culture within 24 hours of collection).

### 3.4 Signs of lower urinary tract disease and urine culture results

Signs of lower urinary tract disease were present in 19 dogs (19/107, 17.8%). When described in the medical record, the most commonly reported signs of LUTD included pollakiuria and inappropriate urination. Of the 15 diabetic dogs with a positive urine culture, 11 dogs (73.3%, 11/15) did not have signs of LUTD present as reported by the owner, therefore 13% (11/88) of dogs with diabetes mellitus fit the definition of subclinical bacteriuria. Of these 11 dogs, 8 dogs had urine collected by cystocentesis and 3 dogs had urine collected from a voided sample but were submitted for culture within 24 hours of collection. Of the 15 dogs with a positive urine culture, 4 dogs had signs of LUTD, therefore only 4% of diabetic dogs (4/107) had evidence of a possible urinary tract infection. Of the 92 dogs with a negative urine culture, 15 dogs exhibited signs of LUTD (16.3%). No association was found between urine culture results and the presence of signs of lower urinary tract disease (OR 1.87; 95% CI 0.59‐6.85, *P* = .46).

### 3.5 Changes in insulin treatment

Out of the 11 dogs that had subclinical bacteriuria, 3/11 (27%) had a change in insulin dosage. The insulin dose was decreased in these 3 dogs because of concern for overregulation of diabetes mellitus because of the findings of suspected seizure‐like activity and intermittent concurrent hypoglycemia events, episodes of collapse shortly after receiving insulin, and intermittent periods of lack of glucose in the urine. The remaining 8 dogs with subclinical bacteriuria (8/11, 73%) had no change in insulin treatment.

### 3.6 Antimicrobial treatment

An antimicrobial was prescribed in 20 dogs (18.7%, 20/107), of which 5 (25%) dogs had signs of LUTD, 12 (60%) dogs had a positive urine culture, and 4 (20%) dogs had both a positive urine culture and signs of LUTD. The most common antimicrobial prescribed was amoxicillin/clavulanic acid (85%, 17/20) followed by enrofloxacin (10%, 2/20) and amoxicillin (5%, 1/20). Three of the 15 dogs (20%, 3/15) with positive urine cultures were not prescribed antimicrobials; however, none of them were found to display signs of LUTD.

### 3.7 Follow‐up information

Of the 11 dogs with a positive urine culture but no signs of LUTD, follow‐up information was available for 10 dogs. All 11 dogs received antimicrobial treatment. Two dogs were given 3 additional courses of antimicrobials of varying duration (10 days‐5 weeks) because of the presence of bacteria in the urine and a positive urine culture. Nocturnal enuresis was later noted in both dogs thought to be consistent with urethral sphincter mechanism incompetence, the previously diagnosed infections were no longer deemed clinically relevant, and no additional courses of antimicrobials were pursued. Three dogs were subsequently diagnosed with subclinical bacteriuria and routine urinalyses were no longer performed and the dogs were reportedly doing well for a follow‐up average of 3 to 7 years. Three dogs were documented to have a negative urine culture after antimicrobial treatment and had no further reports of concern for urinary tract infections. One dog was diagnosed with overcontrol of diabetes mellitus and improved on a lower dose of insulin and had no additional documentation of urinary tract infections. One dog developed seizures thought to be related to poorly controlled diabetes mellitus 17 days after the initial visit and was euthanized. Necropsy did not show histologic evidence of cystitis or pyelonephritis, but a culture of the bladder wall or urine was not obtained.

Of the 15 dogs with signs of LUTD but a negative culture, follow‐up information was available in all 15 dogs. Only one dog received antimicrobial treatment because of the presence of bacteria in the urine and before receiving urine culture results. All of the dogs were re‐evaluated, and no dogs were documented to have a urinary tract infection and were reportedly doing well with an average follow‐up of 3 months to 4 years. Reported reasons for signs of LUTD in these dogs included the following: overflow incontinence because of poorly controlled diabetes mellitus in 6 dogs which resolved with changes to insulin treatment, newly diagnosed diabetes mellitus with overflow incontinence because of severe PU/PD in 5 dogs, newly diagnosed diabetes mellitus with overflow incontinence because of severe PU/PD and concurrent degenerative myelopathy in 1 dog, urethral sphincter mechanism incompetence that was diagnosed several months later in 1 dog, behavioral causes in 1 dog, and suspected cystitis because of previous cystotomy performed 1 month before because of calcium oxalate urolithiasis.

## 4 DISCUSSION

This study demonstrated that although subclinical bacteriuria does occur in diabetic dogs, in contrast to our hypothesis, this was relatively uncommon. Additionally, this study found no association between signs of LUTD and a positive urine culture. Our study did show an association between pyuria and bacteriuria and a positive urine culture, however, it should be noted that a small number of dogs were noted to have pyuria or bacteriuria so future studies with a larger cohort of dogs should be pursued to further support this association. Like previous studies^1^, ^3^, ^13^ these findings suggest pyuria and bacteriuria to be significant predictors of a positive urine culture. This is in contrast however to previous works which have suggested poor or equivocal agreement between urine cytology findings and urine culture results.^1^, ^5^ Our findings suggest that routine urine cultures are not a clinically useful diagnostic tool in diabetic patients, particularly in the absence of pyuria and bacteriuria but further studies are needed to investigate this clinical question.

The findings of pyuria or the cytological presence of bacteria even in the absence of clinical signs can be unsettling to many clinicians. There can be poor agreement between the presence of white blood cells in the urine or the cytologic presence of bacteria and a positive urine culture,^1^, ^2^, ^3^ leading to the recommendation that a urine culture should be submitted regardless of the urine sediment results in diabetic dogs. However, considering the recent definition of SB, the question of whether these dogs had a true UTI and required antimicrobial treatment is unknown. In our study, there was a statistical association between a positive urine culture and pyuria and cytological evidence of bacteria, thus these 2 parameters were the best predictors of a positive urine culture in our diabetic patients. From a proportion standpoint, this is true as dogs with a positive urine culture may or may not have pyuria but in our study, no dogs with a negative urine culture had documented pyuria. Additionally, only one dog with cytologic evidence of bacteria on urine sediment had a negative urine culture, and all dogs with a positive urine culture had bacteria present on urine sediment. However, it should be noted again that this was only observed in a small number of dogs in our study. Additionally, it is important to remember that pyuria and bacteriuria are not an indication to treat with an antimicrobial and findings still need to be taken into consideration of the clinical context.

Some dogs with a negative urine culture were exhibiting signs of LUTD as described by the owner. Only one of these dogs received an antimicrobial but none of the remaining dogs were documented to develop a urinary tract infection or pyelonephritis over a follow‐up period of months to years. There are confounding factors that make the reliance on signs of LUTD difficult to determine in dogs, especially those with diabetes mellitus. Examples include the ability to identify signs of LUTD in dogs with free access to the yard or owners misinterpreting clinical features of disease such as pollakiuria (more suggestive of UTI) and polyuria (common in diabetes mellitus). For example, in our study, the only signs of LUTD that was reported in these dogs was inappropriate urination and was reported because of poorly regulated or newly diagnosed diabetes mellitus in most of the dogs and resolved with initiation of insulin treatment or adjustments in insulin treatment. As a result, a detailed history is particularly important in these patients and performing a routine urine culture in every diabetic patient can potentially result in undue costs to the pet owner, unnecessary use of hospital resources, and potential inappropriate antimicrobial use.

More information is currently needed about the outcomes of treating both diabetic and healthy dogs with subclinical bacteriuria (SB). In our study, 20 dogs were prescribed an antimicrobial, of which 15 dogs had a positive urine culture and 19 dogs had evidence of signs of LUTD. Although our study was retrospective in nature, follow‐up information was available in almost all of the dogs. Although this was observed in a small number of dogs, in the dogs that were treated with an antimicrobial and had a positive urine culture but lacked signs of LUTD, several of these dogs were prescribed additional multiple prolonged courses of antimicrobials that did not appear to affect diabetic control or outcome. Some literature suggests treating SB in animals thought to be at risk for ascending infection such as patients with chronic kidney disease or that are immunocompromised^7^ however, the recent guidelines^8^ suggest that there is a lack of evidence to support treatment of SB in any animal, regardless of comorbidities. This is based on the lack of evidence found in human literature to support treatment of AB in either healthy individuals or those with comorbidities including endocrinopathies^9^ however more prospective studies with outcome measures are also needed in human medicine. Treatment of bacteriuria in humans without clinical signs increases the risk of adverse drug reactions and promotes antimicrobial resistance and often will result in recolonization of bacteria and lack of appropriate bacterial clearance.^9^ This recommendation is for both healthy, and diabetic patients,^9^ and there is no current strong evidence to suggest that dogs should be treated differently from human patients in this regard.

There are limitations to our study. An inherent limitation is because of the retrospective nature of the study design and lack of standardized follow‐up and diagnostics. However, the medical records utilized for this study were obtained from an academic hospital with extensive patient histories. To ensure signs of LUTD were not confused with signs of urinary incontinence at the initial evaluation, any dog with a diagnosis of urinary incontinence, USMI, or was currently on medications for control (diethylstilbestrol, phenylpropanolamine, etc.) of urinary incontinence was excluded. Although polyuria could be considered a sign of LUTD, it was not included in our definition for diabetic dogs as polyuria could be a common manifestation of a poorly regulated diabetic. Another limitation already mentioned is that the presence of signs of LUTD was based on retrospective evaluation of medical records and reliance on the owner to report such observations. Signs of LUTD may have been present but not communicated to the veterinarian writing the medical record. Less likely, signs of LUTD could have been mentioned by the client but not recorded within the medical record or misinterpreted as uncontrolled diabetes mellitus or urinary incontinence. Another limitation of this study because of the retrospective study design is that we cannot make conclusions regarding adverse consequences of not treating a diabetic dog with a positive urine culture. Only 3 dogs with a positive urine culture were not treated with an antimicrobial but they also lacked signs of signs of LUTD. Although we evaluated the follow‐up visits in these 3 dogs and did not find any documentation of clinical complications of not treating over multiple months, the number of dogs not treated was too small to make any meaningful conclusions.

The findings of this study discourage the routine submission of urine cultures in diabetic dogs, particularly when pyuria and bacteriuria are absent but future larger scale studies are needed. Urine cultures are expensive and likely impose an unnecessary financial burden on clients so the decision to submit a urine culture should be taken into consideration of the clinical context of the patient and urine cytologic findings.

## CONFLICT OF INTEREST DECLARATION

Authors declare no conflict of interest.

## OFF‐LABEL ANTIMICROBIAL DECLARATION

Authors declare no off‐label use of antimicrobials.

## INSTITUTIONAL ANIMAL CARE AND USE COMMITTEE (IACUC) OR OTHER APPROVAL DECLARATION

Authors declare no IACUC or other approval was needed.

## HUMAN ETHICS APPROVAL DECLARATION

Authors declare human ethics approval was not needed for this study.
